# Mild cognitive impairment and *f*MRI studies of brain functional connectivity: the state of the art

**DOI:** 10.3389/fpsyg.2015.01095

**Published:** 2015-08-04

**Authors:** Laia Farràs-Permanyer, Joan Guàrdia-Olmos, Maribel Peró-Cebollero

**Affiliations:** ^1^Departament de Metodologia de les Ciències del Comportament, Facultat de Psicologia, Universitat de BarcelonaBarcelona, Spain; ^2^Institut de Recerca en Cervell, Cognició i ConductaBarcelona, Spain

**Keywords:** mild cognitive impairment, *f*MRI, connectivity, statistical analysis, review

## Abstract

In the last 15 years, many articles have studied brain connectivity in Mild Cognitive Impairment patients with fMRI techniques, seemingly using different connectivity statistical models in each investigation to identify complex connectivity structures so as to recognize typical behavior in this type of patient. This diversity in statistical approaches may cause problems in results comparison. This paper seeks to describe how researchers approached the study of brain connectivity in MCI patients using fMRI techniques from 2002 to 2014. The focus is on the statistical analysis proposed by each research group in reference to the limitations and possibilities of those techniques to identify some recommendations to improve the study of functional connectivity. The included articles came from a search of Web of Science and PsycINFO using the following keywords: *f* MRI, MCI, and functional connectivity. Eighty-one papers were found, but two of them were discarded because of the lack of statistical analysis. Accordingly, 79 articles were included in this review. We summarized some parts of the articles, including the goal of every investigation, the cognitive paradigm and methods used, brain regions involved, use of ROI analysis and statistical analysis, emphasizing on the connectivity estimation model used in each investigation. The present analysis allowed us to confirm the remarkable variability of the statistical analysis methods found. Additionally, the study of brain connectivity in this type of population is not providing, at the moment, any significant information or results related to clinical aspects relevant for prediction and treatment. We propose to follow guidelines for publishing fMRI data that would be a good solution to the problem of study replication. The latter aspect could be important for future publications because a higher homogeneity would benefit the comparison between publications and the generalization of results.

## Introduction

In recent years, numerous papers have been published on brain connectivity, a key element to understand brain functioning (for example, Cole et al., 2010; Bai et al., 2011; Binnewijzend et al., 2012; Zanto et al., 2014). Brain connectivity has raised great interest in the field of quantitative and computational neuroscience. Computational neuroscience is devoted to identifying new tools for analysis, mathematical, and statistical modeling, and computational resources to deal with brain signal data in any modality.

Currently, several approaches and techniques have been used to quantify the different types of brain connectivity. In the present paper, we focused on those derived from functional Magnetic Resonance Imaging (*f*MRI): structural connectivity, functional connectivity, and effective connectivity. The reason for this selection lies in the fact that most of the existing papers focused on those three concepts of connectivity. Structural or anatomical connectivity is defined as the set of physical connections between neuronal units. The physical model of anatomic connections is relatively stable in short time periods, but hardly stable in the long term due to the morphological modifications caused by brain plasticity (Symms et al., 2004). In fact, structural MRI has become the accepted standard for routine examination of the brain, offering exquisite anatomical detail and high sensitivity to pathological changes (Symms et al., 2004). On the other hand, functional connectivity is essentially a statistical concept that studies remote neuronal network relationships that show a certain interrelation (Friston et al., 2014). Fluctuations in the blood oxygenation level-dependent (BOLD) signal in functional connectivity may present a valuable data resource for delineating the human neural functional architecture (Cole et al., 2010). On the basis of biological considerations, functional imaging can be regarded as the method that provides dynamic physiological information, whereas structural imaging provides static anatomical information (Symms et al., 2004). Lastly, effective connectivity addresses the direct influence of a brain region on the physiological activity registered in other brain regions (Friston, 1994; Friston et al., 2003). Anatomical imaging is obtained through the study of brain hydrogen and oxygen, and through the diamagnetic changes produced during a task that compromises some cognitive cost, even in a resting state (for more information, see Ogawa et al., 1990; Logothetis et al., 2001; Buxton, 2002; Huettel et al., 2009).

In recent years, there has been a growing interest in studying connectivity through the resting states. Resting-state functional MRI is an imaging method that reflects synaptic activity through changes in blood fluctuations and the oxyhemoglobin: deoxyhemoglobin ratio (Schölvinck et al., 2010; Binnewijzend et al., 2012). In this type of study, the participants may not move at all while they remain with their eyes closed without thinking about anything in particular for a specific period of time. This method permits the investigation of spontaneous activity, and the analysis of the spatiotemporal coherence of fMRI activity reveals several distinct domains of correlated activity in the brain (Schölvinck et al., 2010). The resting state allows us to minimize the amount of noise of the images obtained, given that the participants conduct no activity whatsoever and no stimulus is presented.

A strong debate has emerged of late about the best way to model functional connectivity network estimations, given the crucial role of the statistical model (Gates et al., 2010). The different options in terms of connectivity analysis techniques became clear when the authors mentioned and described four different approaches for connectivity mapping. These approaches were Structural Equation Modeling (SEM) which seemed to appear the most straightforward application, and also the most common, in connectivity estimations. In SEM, covariance patterns of contemporaneous BOLD time series illustrate brain functional connectivity via directed pathways (McIntosh and Gonzalez-Lima, 1994). Another approach was the Dynamic Causal Modeling (DCM) that uses deterministic differential equations to assess how regions relate and estimate external modulation of connections (Friston et al., 2003). The next approach was the vector autoregression (VAR), which estimates the influence that data from ROIs at previous time points have on a given ROI's BOLD activity. Finally, the authors presented an improved version of the unified SEM approach of Kim et al. (2007) to model contemporaneous and sequential relationships among ROIs (Gates et al., 2010), but they recorded the statistical analysis of this type of data.

Other methods of functional connectivity analysis that is important to mention are Granger Causality and the studies of dimensionality. Granger causality is a statistical method for assessing directional influences between simultaneously time series (Zhou et al., 2009). These directions and magnitudes of Granger causality are interpretable in terms of the directions and magnitudes of synaptic transmissions between different neurons and brain areas (Brovelli et al., 2004). On the other hand, the studies of dimensionality allow us to reduce the amount of data to analyze and thus facilitate its later analysis. It is typical to have a large amount of voxels available to conduct the analyses of this type of data. For this reason, the approaches that allowed us to reduce the amount of data while remaining independent between them, as in the case of Independent Component Analysis (ICA), which is the most typical method in this category, are very frequent in this context. In fact, reduction techniques are fundamental to establish reasonable statistical models because the use of ROIs seems logical and sensible.

Therefore, as an especially relevant aspect, we will focus on the different approaches to the study of functional connectivity, which will be itemized below. The images obtained in the study of functional connectivity (*functional Magnetic Resonance Imaging—f*MRI) allowed us to anatomically and functionally locate the different cognitive processes based on the increase in blood flow and neuronal activity (Bandettini et al., 1997).

Different possibilities exist when choosing the focus of the study of functional brain connectivity. Authors often opt for a particular sampling population. In some cases, the authors intended to study the connectivity of individuals suffering from a specific pathology, mostly to check whether and how that condition is reflected in connectivity. In the last decade, the population of adults suffering from Mild Cognitive Impairment (MCI) has been often chosen from among these subjects. We recognize MCI as the mild but defined deterioration in relation to the previous cognitive performance, confirmed by an observer and clinically quantified by neuropsychological tests (Flicker et al., 1991; Mueller et al., 2005). MCI has been studied on numerous occasions as the decline in cognitive functions between normal aging and Alzheimer's or other types of dementia (Dickerson et al., 2005). It has also been proven that MCI patients have a higher risk of Alzheimer's than those with no such cognitive alterations (Machulda et al., 2003; Han et al., 2011). MCI patients form a remarkably heterogenic group due to the diverse symptomatology they can present (Celone et al., 2006). Additionally, there are different levels of deterioration in MCI, which presents: participants in an early stage of illness could clearly show different connectivity patterns from those in an advanced stage (Machulda et al., 2003).

Brain connectivity in MCI patients has been widely researched recently. The complexity in designing a study on this topic lies not only in the heterogeneity of the disorder, but also in the difficulty of choosing the most valid statistical approach to analyze the *f*MRI data. This consideration is very important in classical approaches of clinical studies based on the comparison between groups and performance analysis, but it is more relevant if we defend the hypothesis that one particular connectivity structure can show more profound characteristics of MCI patients. Therefore, this current stage where statistical models that estimate connectivity networks are varied and, sometimes, incomparable, make it extraordinarily difficult to know the complexity of connectivity networks in detail. This specificity is not exclusive of the MCI-diagnosed population, but it has a particular relevance in this case due to the relevance of this disease and to its future increased presence because of increased life expectancy.

The choice of MCI to study functional brain connectivity is determined by the fact that it is one of the few pathologies to show brain changes at a structural level, which usually occurs in neurodegenerative disorders. For example, the decrease in the size of the hippocampus and the high atrophy in the medial temporal lobe, as well as the loss of the volume of gray matter in the frontal and parietal regions, are structural changes present in this disorder (Mueller et al., 2012). This distinctive feature makes MCI of great interest, given that we intend to study whether there is functional correspondence in such structural changes and what type it is.

In addition, from a clinical perspective, the study of MCI patients revealed a special interest of certain very important aspects. In the first place, the crucial goal is to determine those variables which would be appropriate predictors of a negative course of the pathology. Thus, it is essential to know that certain elements might indicate that one person with an MCI diagnosis could change from one domain (memory deficits) to multiple domains (language, working memory, etc.) or even develop irreversible dementia. Identifying whether the interaction between tasks, especially those connected to mnemonic performance, and connectivity networks in fMRI might be an option to predict the prognosis (Sandry and Sumowski, 2014). Likewise, a long tradition exists in the study of the effects of Cognitive Reserve (CR), according to the definition by Jones et al. (2011) in the prediction of MCI severity and evolution. Apart from the difficulty of implementing this latent variable (Lojo-Seoane et al., 2012, 2014), it is true that the analysis of possible links between CR and the connectivity network, especially in the Default Mode Network (DMN), should provide some evidence on the matter. The DMN is an extensively known connectivity network involving several brain areas, is remarkably affected in these patients and is also widely studied. Finally, due to the fact that it is a disease related to elderly populations, it seems necessary to determine whether estimated connectivity networks in different tasks might be an indication of risk or frailty in elderly people. This needs to be kept in mind for risk prediction (Sumowski et al., 2014). We must keep in mind the most clinical and applied aspects of the study of connectivity in fMRI paradigms, as it should provide further and deeper evidence on the course of the pathology.

Methodological characteristics and differences between *f*MRI studies were reported recently by Carp (2012). He explained the flexibility of the 241 articles consulted in terms of methodological details, such as the experimental design, data acquisition, and data analysis. The author noted too many differences in analytic procedures between articles. Finally, he concluded that this high level of analytic flexibility could be a risk factor for bias in scientific research (Carp, 2012), and he advised the use of the guidelines provided by Poldrack et al. (2008) for reporting *f*MRI data.

As mentioned above, there is a great diversity of statistical approaches for the study of the connectivity models in the MCI population, which makes it difficult to extract conclusions based on different authors. As a consequence of the above described situation, the aim of this paper was to facilitate an in-depth analysis of the mechanisms for statistical modeling used to estimate connectivity in MCI. We will also extract some considerations on the use of some modeling techniques or others in order to assess their advantages and disadvantages.

Additionally, we will try to analyze whether this type of study has provided some evidence on the possibility that connectivity networks in fMRI allow us to further know the role of specific clinical variables in the course of MCI.

## Materials and methods

### Article search

The databases used to conduct the article search were PsycINFO and Web of Science. In order to be included in the present study, the articles were required to comply with the following criteria: (a) be original works whose goal was to study functional connectivity through *f*MRI in persons with MCI and (b) had to explain the type of analysis of functional brain connectivity applied, as well as the results obtained from it. For these reasons, the key words used to conduct the search were *f*MRI, MCI, and Functional Connectivity. After a preliminary search with the aforementioned key words, we located 81 articles, two of which were discarded because they did not include a detailed analysis of the sample's functional brain connectivity. Then, all the selected articles contain connectivity models and statistical analysis information. Meta-analyses and reviews with those keywords were selected, but its information appears only in the descriptive sections of this document to avoid repeating information in the other sections. Thus, 79 articles were included in the present paper (identified with * in the bibliography) which contained more than one type of approach to the analysis of functional brain connectivity. For consistency, the search was replicated by two independent researchers. They obtained 100% agreement on the selected articles.

### Article assessment

For each paper selected, we analyzed specific sections in relation to the study of MCI with *f*MRI. Only the information given in the articles was assessed (no author was contacted to obtain further information). The necessary elements to comprehend the followed procedure in each case were selected for the functional connectivity analysis. For each paper, we assessed the goal of the research, the type of task the participants were required to do (including resting state and all types of tasks), the brain areas involved, the use of regions of interest (ROI) analysis, the connectivity estimation model used, and the data analysis techniques applied. In the results section, we summarized the main results for each section. Those results can be seen in detail for each study included in Table 1.

**Table 1:** **Detailed results of selected articles in the survey**.

**Authors (Year)**	**Connectivity estimation model/ statistical analysis**	**Survey/conclusions**
Li et al., 2002	COSLOF index Two-sample *t*-test	Low COSLOF index may reflect dysfunctions in functional synchrony in MCI and AD COSLOF index can make out AD, MCI, and controls. Possible biomarker for decline
Machulda et al., 2003	Correlations ROC curve analysis	More activation in codification areas by adults with preserved cognition than MCI Absence of statistical differences between MCI and AD
Dickerson et al., 2004	Boxcar function Image contrasts	More impaired hippocampus participants activate a bigger parahippocampal area than less impaired
Greicius et al., 2004	ICA Best-fit component Two-sample *t*-test	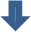 DMN, hippocampus DMN connectivity changes could be a biomarker for cognitive impairment
Johnson et al., 2004	GLM Temporal autocorrelation with Regression Algorithm REA One-sample and two-sample *t*-test	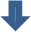 Temporal lobe (R) Importance of Hippocampus activity in *f*MRI MCI
Dickerson et al., 2005	Boxcar functions ANOVA Partial Correlations Pearson Coefficient Correlations	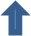 Hippocampus Increased hippocampus activation in associative memory tasks could be a biomarker for future MCI or AD
Rombouts et al., 2005	MEA Regression estimation parameters	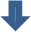 DMN, Frontal Anterior Initial phase of DMN activation/deactivation seems to be a possible biomarker
Bokde et al., 2006	LinealCorrelation Coefficient Fisher's Z transformation	The presence of Alzheimer's neuropathology in MCI affects functional connectivity from right fusiform gyrus to visual areas and medial frontal areas Compensatory processes in parietal lobe
Celone et al., 2006	ICA One-sample and two-sample *t*-test	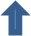 Hippocampus, neocortical areas 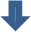 Parietal and Medial areas, DMN Non-linear trajectory in AD prodromal course
Johnson et al., 2006	High-pass filtering (128) Temporal autocorrelations REA One-sample and two-sample *t*-test	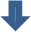 Temporal Inferior lobe Efficiency decrease in learning temporal ventral system
Krishnan et al., 2006	GLM Two-sample *t*-test REA Correlations	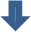 Frontal lobe, MTL More extended activation, possible compensatory mechanism
Hämäläinen et al., 2007	One-sample and Two-sample *t*-test Correlation SVC at detected peak coordinates	Compensatory mechanisms in MCI 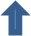 Cuneus, sulcus intra-parietal, and intra-occipital 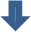 Cingulate
Kircher et al., 2007	High-pass filtering (1/128) GLM One-sample and two-sample *t*-test	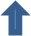 MTL anterior, Hippocampus MCI patients need more sources to solve the task
Sperling, 2007	Review	Hippocampus and Prefrontal cortex are critical for successful memory MCI have a phase of increased connectivity, compensatory mechanisms
Teipel et al., 2007	FEA GLM Pearson Coefficient Correlation	Functional connectivity divergence between ventral and dorsal visual systems in MCI and AD, related with neuronal density
Vannini et al., 2007	*t* contrasts Boxcar function Orthogonal predictors	MCI converters to AD have functional connectivity alterations but not performance alterations 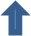 Parietal lobe
Wang et al., 2007	Correlations Fisher's Z transformation One-sample and two-sample *t*-test Seed-reference correlations	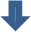 Connectivity between frontal and parietal 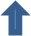 Connectivity between prefrontal and other areas Disconnections between anterior and posterior brain areas, but increased connectivity inside lobes
Bai et al., 2008	Kendall's concordance coefficient (*W*)	MCI compensation mechanisms in limbic system 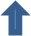 Inferior Parietal lobe (R), Fusiformgyrus (R), Putamen
Bokde et al., 2008	GLM MEA Random Field Theory correction One-sample *t*-test	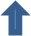 Frontal lobe Heterogeneity of MCI
Dickerson and Sperling, 2008	Review	Varied results. Heterogeneity in MCI connectivity patterns *f*MRI seems optimum for diagnostic, symptoms severity and memory abilities
Kaufmann et al., 2008	FEM REA	Inhibitory control deficit Compensatory mechanisms
Miller et al., 2008	REA	Hippocampus *f*MRI images could be a biomarker for cognitive decline Hyper-activation as a compensatory mechanism
Trivedi et al., 2008	FDR in multiple comparisons	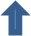 Frontal Inferior cortex (L) 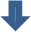 Parahippocampalgyrus, Frontal Medial cortex
Zhou et al., 2008	ICA	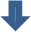 Cingulate Posterior cortex, Hippocampus Directly related with cognitive impairment
Clément and Belleville, 2009	Overlap Ratios Jaccard coefficients	Less activation in 2nd session than 1st No MCI repercussion in *f*MRI reliability, but more secure in group analysis than individual
Jauhiainen et al., 2009	Mann–Whitney Coefficient (*U*) Spearman Correlation	Entorhinal cortex seems better than Hippocampus for clinical classification (MCI/AD)
Machulda et al., 2009	One Sample *t*-test	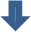 Activation in aMCI and naMCI than cognitive preserved No statistical significative differences between aMCI and naMCI, but aMCI seems to have less activation on multimodal association cortical areas
Mandzia et al., 2009	Two-sample *t*-test Correlation	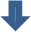 Prefrontal Inferior Complex relationship between activation in impaired areas and task performance Difficulties because of MCI heterogeneity
Pihlajamäki and Sperling, 2009	High-pass filtering (140.0) Temporal series with autocorrelation correction	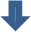 Posteromedial lobe Deactivation pattern progressively impairing while the memory impairment goes on APOE e4 carriers are more impaired than non-carriers
Poettrich et al., 2009	ANOVA One-sample and two-sample *t*-test GLM	Alteration in neural mechanisms of long term memory retrieval, episodic, semantic, and autobiographical
Solé-Padullés et al., 2009	ANOVA *X*^2^ Partial Correlations Two-sample *t*-test GLM	Inverse effect between Cognitive Reserve and functional connectivity in MCI and AD Adults with preserved cognition: high CR, high efficiency (less activation) MCI and AD: low CR, low efficiency (more activation)
Woodard et al., 2009	AUC SEA ANOVA	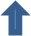 Parietal Posterior lobe, Temporal Lobe Association name task seems to be an optimum task for cognitive decline as a biomarker
Agosta et al., 2010	Regression *t*-test ANCOVA SVC	Decreased hippocampal volume seems to be compensated by cortex increased connectivity Functional correlates of AD and MCI in MTL and DMN
Frings et al., 2010	GLM REA ANCOVA and ANOVA t contrasts SVC for multiple comparisons	Precuneus and Cingulate Posterior cortex connectivity showed alterations Finding alterations in those areas seemed a good predictor for future decline
Gold et al., 2010	Deconvolution Analysis GLM One-sample *t*-test	Neocortical alterations 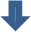 Fusiform Medial gyrus, MTL
Kochan et al., 2010	GLM One-sample *t*-test REA ANOVA *d'* Performance measure	Activation differences between low and high MCI load 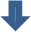 Precuneus, Anterior cortex (low) 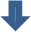 Precuneus, Cingulate Posterior and Medial (high)
Qi et al., 2010	ICA PCA Fisher's Z transformation	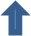 Frontal Superior gyrus, Prefrontal Medial cortex, Parietal Inferior lobe, Medial Temporal gyrus 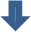 Cingulate Posterior cortex, Parietal Inferior lobe
Sala-Llonch et al., 2010	Tensorial ICA Dimensionality vectors Pearson Coefficient Correlation Gaussian/gamma Mixture Model	Two visual Networks were identified MCI presented visual connectivity changes, especially in dorsal way, with compensatory mechanisms
Yassa et al., 2010	Behavioral vectors Deconvolution Analysis GLM ANOVA	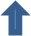 Hippocampus (CA3 region) CA3 hippocampus region seemed to be the base of neural deficits in episodic memory tasks of amnesic MCI Changes in CA3 activation patterns as a possible biomarker for future decline
Bai et al., 2011	Correlation Fisher's Z transformation	Changes in hippocampus subregional networks could be an early indicator for disfunction
De Rover et al., 2011	Two-sample *t*-test SVC FDR in multiple comparisons REA	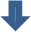 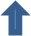 Hippocampus Confirmed importance of MTL in visuospatial tasks
Hampstead et al., 2011	REA GLM Correlation-purged GCA Monte Carlo simulation	Frontal Medial, Parietal, Occipital cortex changes after training 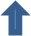 Connectivity in the whole brain, in general
Han et al., 2011	ALFF &fALFF	DMN shows significant differences in LFO in MCI
Lenzi et al., 2011	*t*contrasts	MCI in early stages develop compensatory mechanisms. Absence of those mechanisms in advanced MCI
Petrella et al., 2011	ICA GOF	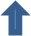 GOF mean in MCI converters (Cingulate Posterior, Precuneus, Parietal inferior lobe)
Protzner et al., 2011	ICA PLSA	More brain regions than usual must be activated to solve the task
Wang et al., 2011	Correlations Fisher's Z transformation One-sample and two-sample *t*-test REA Monte Carlo correction	Hippocampus-cortex connectivity system is altered in MCI Hippocampus connectivity shows differences 3 years after, illustrating the impairment process and evolution
Baglio et al., 2012	One Sample *t*-test ANOVA Multiple regression	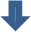 Temporal areas connectivity Compensatory mechanism in frontal regions could supplement the decay of part of neural circuit
Binnewijzend et al., 2012	ICA Laplace approximation Dual regression approach between subjects	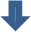 DMN, Parietal Lateral cortex MCI is between AD and controls in the results
Clément and Belleville, 2012	REA ANCOVA One-sample and two-sample *t*-test	Hyper-activation in most impaired areas as a compensatory mechanism
Han et al., 2012	Fisher's Z transformation FDR in multiples comparisons Partial correlations	Correlation between episodic memory and processing speed 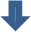 Frontal Orbital and Central (R), Putamen (L), Caudate (R), Temporal Superior (L), Cingulate Posterior (R) 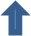 Fusiform (L), Frontal Inferior (R), Pre-central (L)
Jin et al., 2012	Spatial ICA MDL dimensionality estimation PCA One-sample and two-sample *t*-test	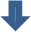 Parietal Posterior cortex, MTL, Prefrontal Lateral cortex, Medial Temporal gyrus 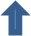 Parietal Inferior lobe, Prefrontal Medial cortex, Cingulate Medial cortex *f*MRI restings tate as an important biomarker for cognitive impairment
Liu et al., 2012	SWA Node Analysis	Topological abnormalities in MCI and AD connectivity patterns in all brain networks
Mueller et al., 2012	Review	MCI: alterations in brain activity during visual processing and working memory 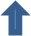 Temporal Medial lobe, Hippocampus 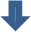 Temporal Medial lobe, Cingulate Posterior cortex, Parietal lobe Increased activation in Hippocampus seemed a predictor of cognitive decline Hippocampus volume could be a predictor for MCI converters to AD
Staffen et al., 2012	Contrast images between conditions One-sample and Two-sample *t*-test Fisher's Z transformation Correlation	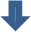 Temporal lobe, Temporal gyrus, Temporal superior sulcus, Cuneus (L), Cingulate Anterior cortex, Frontal gyrus
Wang et al., 2012	GLM Correlations Fisher's Z transformation One-sample and two-sample *t*-test Monte Carlo simulation	Cingulate Posterior cortex alterations are very present in MCI Alterations in connectivity between Cingulate Posterior cortex and other regions of DMN
Wee et al., 2012	Deformation fields estimation Frequency-band division Pearson Coefficient Correlation One-sample *t*-test	fMRI and DTI techniques provide valuable information Both techniques are complementary
Zhang et al., 2012	Regional Homogeneity ANOVA Two-sample *t*-test	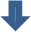 DMN DMN, especially Cingulate Posterior cortex, has an important role in memoristic network
Alichniewicz et al., 2013	Two samples *t*-test Boxcar functions Regression GLM ANOVA	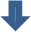 Inhibition functions of anti-saccadic movements The alteration of anti-saccadic movements might reflect early AD
Browndyke et al., 2013	Meta-analysis	Variations in applied paradigms make it difficult to extract inferences from the results of the review 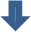 Parahippocampal gyrus, Entorhinal volume Abnormal connectivity pattern in DMN 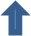 Prefrontal lobe
Clément et al., 2013	GLM REA ANOVA	MCI high cognition: more activation (compensatory mechanism) MCI low cognition: less activation
Faraco et al., 2013	FEM contrasts MEA Markov Chain Monte Carlo sampling	Important role of Lateral Temporal lobe in MCI detection. Possible biomarker of MCI
Graewe et al., 2012	*d'* Performance mesure GLM ANOVA LDA REA	Aberrant pattern activation in Fusiform face area and Occipital face area. Possible biomarkers for cognitive decline
Hahn et al., 2013	ICA PCA Fisher's Z transformation One-sample *t*-test ANOVA	Intrinsic brain networks are impaired in MCI and AD Structural connectivity is reduced in MCI. They convert to AD in 3 years
Parra et al., 2013	Standard GLM High-pass filtering Serial Correlation with autoregression REA Two-sample *t*-test ANOVA SVC	Absence of improved performance in emotional memory task in MCI and AD 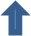 MTL, Frontal lobe
Smith et al., 2013	Deconvolution Analysis GLM AUC calculation	Exercise intervention seems to increase the capacities of MCI patients and adults with preserved cognition capacities More efficiency in neural networks
Wang et al., 2013	ICA GLM ANCOVA and ANOVA *X*^2^	DMN involved in episodic memory processing DMN alterations as a possible biomarker for MCI converters to AD
Yao et al., 2013	Pearson Correlation Coefficient Fisher's Z transformation Two-sample *t*-test	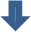 DMN, Amygdala
Zamboni et al., 2013	GLM	Prefrontal medial cortex and Temporal anterior lobe seem to be related with self-awareness, especially in AD
Zhou et al., 2013	Gaussian Random Field Theory Spearman and Pearson Correlation Coefficient	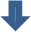 DMN Significant correlation between *f*MRI data and fALFF MCI compensation mechanisms Vascular, functional and pathological measures: optimum to predict AD conversion
Dunn et al., 2014	Pearson Correlation Coefficient Fisher's Z transformation Bivariate Regression Two-sample *t*-test Dunn and Clark Statistic (*Z*_*i*_)	Disconnection between hippocampus and cingulate posterior cortex in amnesic MCI Non amnesic MCI can integrate DMN information
Haller et al., 2014	Tensorial ICA GLM ANOVA FEM	Posterior displacement of working-memory brain activation patterns after caffeine administration Compensatory mechanism to counterbalance a frontal lobe disfunction
Liang et al., 2014	Correlation-purged GCA	Connectivity alterations independently from gray matter atrophy 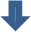 Hippocampus (R), Fronto-Parietal Control Network 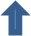 Hippocampus (L), Frontal (R)
Puente et al., 2014	Two-sample *t*-test ANCOVA	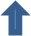 Orbitofrontal cortex, Parietal Posterior cortex
Wee et al., 2014	Pearson Correlation Coefficient Sparse regression with and without group constraint via l_i_-norm regularization	A novel approach to infer functional connectivity networks is proposed New approach seems capable in construction functional connectivity network that yields improved classification compared with Pearson Correlation Coefficient
Yao et al., 2014	Pearson Correlation Coefficient Fisher's Z transformation One sample and Paired *t*-test Monte Carlo Simulation	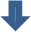 Amygdala connectivity Changes in Amygdala connectivity could be a potential marker of preclinical MCI
Zanto et al., 2014	*t*-test Test-retest analysis ANOVA Intraclass correlation coefficient	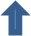 Reliability in cortex activations 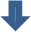 Reliability in subcortical regions activation A delayed recognition task with minimum 30 trials per condition would produce better reliability in regions susceptible to change in MCI
Zhou et al., 2014	ANOVA and ANCOVA *X*^2^ ALFF	Changes in ALFF in diabetes patients in Frontal lobe, Temporal lobe, Hippocampus, Amygdala and Precuneus during resting-state Less pronounced alterations in MCI without Diabetes
Zhu et al., 2014	DICCCOL *t* contrasts Correlation-based feature selection	Connectome signatures showed high accuracy in MCI and control classification and differentiation Connectome scale seemed a possible biomarker

FDR, False Discovery Rate; GCA, Granger Causality Analysis; MEA, Mixed Effects Analysis; ICA, Independent Component Analysis; GOF, Goodness of fit; SVC, Small Volume Correction; GLM, General Lineal Model; REA, Random Effects Analysis; SWA, Small World Analysis; ALFF, Amplitude of Low Frequency Fluctuations; fALFF, fractional Amplitude of Low Frequency Fluctuations; MDL, Minimum Description Length; AUC, Area Under the Curve; FEM, Fixed Effects Model; LDA, Linear Discriminant Analysis; PLSA, Partial Least Squares Analysis; SEA, Spatial Extent Analysis; COSLOF, Cross-correlation coefficients of spontaneous low frequency; DICCCOL, Dense Individualized and Common Connectivity-based Cortical Landmarks; PCA, Principal Component Analysis

### Sample characterization

Numerous journals have published articles analyzing the functional brain connectivity of MCI patients. *Neurobiology of Aging, Journal of Alzheimer's Disease, NeuroImage*, and *Human Brain Mapping* are the journals with the highest number of studies published included in the present paper.

Table 2 summarizes the journals from articles were taken for analysis.

**Table 2:** **Summary of Journals in the survey**.

**Journal Title**	**Number of articles**
Neurobiology of Aging	8
Journal of Alzheimer's Disease	8
Human Brain Mapping	7
NeuroImage	7
Neuropsychologia	4
Neurology	3
Dementia and Geriatric Cognitive Disorders	3
Psychiatry Research	3
Brain	3
Cortex: a journal devoted to the study of the nervous system and behavior	3
Alzheimer's and Dementia: the Journal of the Alzheimer Association	2
Journal of Neurology, Neurosurgery and Psychiatry	2
Journal of the International Neuropsychological Society	2
PloS One	2
Radiology	2
Other	20

Journals such as the American Journal of Neuroradiology, Frontiers in Psychology, Journal of Magnetic Resonance Imaging, or European Journal of Radiology have published one article included in the present paper. All journals with one paper selected are included in the *Others* category.

**Figure 1:**
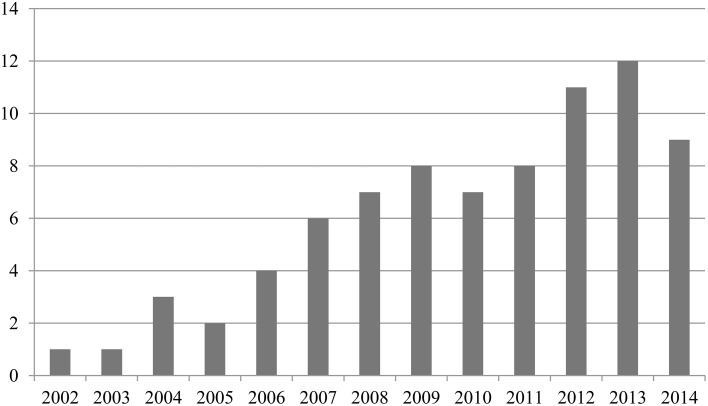
**Summary of publication years in the survey**.

The articles selected were published between 2002 and 2014, although most of them were published between 2007 and 2013. No articles were found before 2002 with the selected keywords. The exact number of articles by year of publication appears in Figure 1.

## Results

The goal of the majority of the papers included was to compare the functional brain connectivity of MCI patients to the functional brain connectivity of Alzheimer's patients or that of adults with no cognitive deterioration. Accordingly, the different studies set forth different types of tasks—-detailed in the following section—in order to study the different connectivity patterns in one or several brain regions—which will also be explained below. In some cases, these regions were defined previously in the aims, and in others, they were mentioned afterwards according to the results obtained.

In some cases, the researchers intended to study the properties of *f*MRI imaging in relation to its reliability or the reproducibility of the data. Other studies compared *f*MRI to other types of brain connectivity data, such as PET imaging (Positron Emission Tomography). Lastly, the goal of some authors was to find or suggest possible biomarkers for the detection of MCI in the early stages of the disorder, which would entail a remarkable breakthrough in treatments and therapeutic interventions for these patients. It is important to note that some studies covered more than one of the goals discussed above, such as the study of brain functioning and, at the same time, presented the possibility of finding biomarkers for the early detection of AD.

Table 3 summarizes the main goals defined in the selected papers.

**Table 3:** **Summary of research goals, use of ROI analysis, brain regions, and statistical analysis in the survey**.

		**Number of articles**
Investigation goals	Compare brain activity between MCI and Alzheimer's and/or elderly with preserved cognition	64
	Properties and characteristics of *f*MRI/Comparison with other types of signals	9
	Find biomarkers for early MCI detection	7
	Others	3
Tasks and cognitive paradigms	Resting state	25
	Face-encoding task	6
	Face-name match task	5
Brain region	Hippocampus and hippocampal gyrus	36
	Inferior parietal lobe and cortex	33
	Parahippocampus and parahippocampal gyrus	27
	Posterior cingulate cortex and gyrus cingulate	23
	Precuneus	23
	Prefrontal cortex	17
	Fusiform	17
Use of ROIs Analysis	Hypothesis driven	27
	Data driven	19
	Absence of ROIs analysis	29
Hippocampus as a ROI	Hypothesis driven	17
	Data driven	5
Connectivity model/Statistical Analysis	Classic parametric strategies	63
	General lineal model approximations	22
	Dimensionality study models	12
	Specific techniques of Model's fitting	24

*When more than one type of statistical analysis within the same group was used in one paper, the frequency was one for this category. In cases in which different categories of analyses were used, the frequency was one for each category used*.

The tasks chosen by the research teams to study the functional connectivity of MCI patients varied. Many studies opted for memory tasks, mainly semantic or episodic memory, although in some cases, they also used working memory, associative memory, or even emotional memory. Most of the studies with memory tasks presented experiments with different phases in which the data obtained in the information codification phase was distinguished from the data obtained in the recovery phase. In some cases, information fixation phases were also present, as well as neutral or basal phases. This situation reflects the need to evaluate this type of patient in memory domains related to this pathology. It is important to keep in mind that MCI diagnoses always involve the presence of subjective complaints of memory mistakes and, therefore, the generation of cognitive tasks and paradigms is strongly related to common components of memory models.

However, most of the authors decided to apply a resting state paradigm in their studies. As it was explained previously, resting state allows investigating spontaneous activity, and permitting to minimize the noise of the images obtained.

Other types of tasks included problem solving, empathy tasks, sound differentiation, and visual-spatial attention tests. Most of these tasks were visual, although we could find some listening or verbal tasks. We also found papers that combined different types of tasks, or in which more than one task was conducted. More specifically, the resting state was the task most frequently asked of participants, followed by face encoding and face-name matching tasks. The frequencies of such tasks can be seen in Table 3.

The brain regions in which functional connectivity was studied varied. Some researchers suggested studying the whole encephalon, while others preferred to focus research on some specific areas.

The studies frequently focused on the brain regions of the Medial Temporal Lobe (MTL), because MCI patients often present alterations in this connectivity network. Within this lobe, the hippocampus and the regions adjacent to it appeared in almost every paper analyzed. According to the mentioned presence of disease at the DMN in these patients, we found many papers remarking on the changes in connectivity patterns in different areas within the DMN network.

Table 3 shows the brain areas that appeared most frequently in the papers analyzed, disregarding review, or meta-analysis articles, which were excluded in this table because most of the articles in those reviews and meta-analysis were included in our review and we sought to avoid repeated information.

The analysis of the Regions of Interest (ROIs) appears frequently in the papers selected. The most frequently selected ROI is the hippocampus, although often we can also find other regions of the Medial Temporal Lobe (MTL), as well as those areas included within the DMN, such as the ventro- or dorsomedial prefrontal cortex, the retrosplenial and posterior cingulated cortexes, the inferior parietal lobe, and the hippocampus (including the entorhinal and parahippocampal cortexes) (Buckner et al., 2008).

Table 3 shows the number of articles that opted for the analysis of ROIs defined prior to the data collection (Hypothesis Driven); it also shows those that conducted it after an early analysis based on the detection of activated areas (Data Driven). Additionally, it shows those that did not use this type of approach. Review or meta-analysis articles were disregarded. It can be seen that most of the authors chose to conduct the analysis of ROIs, especially the analysis of regions defined prior to the data collection (Hypothesis Driven). However, the Data Driven choice was also made by many research teams. Nevertheless, this description does not contribute to the choice of the statistical model. Due to the diversity in statistical resources employed, it was difficult to identify comparable results.

### Connectivity estimation model and data analysis

As mentioned early in this paper, the authors made numerous proposals to analyze *f*MRI, and they used a wide variety of models to estimate functional connectivity. In order to organize the different types of analyses and approaches, we decided to put forward a descriptive classification of the different types of models and to do so in four large groups: (i) Classic parametric strategies; (ii) Approaches based on the general linear model; (iii) Studies based on models pertaining to the study of dimensionality; and finally (iv) techniques based on fitting specific models. This classification was generated with the unique proposal of arranging the different approaches and techniques used through a significantly recognizable group. Then, we tried to facilitate the ordering of statistical models that were often used in this field.

The classic parametric strategies were the most used by researchers in this field. Within this group, the authors chose a large number of different analyses, among which we found, for example, correlations, partial and semi-partial correlations, Kendall's coefficient of concordance, *t* tests, ANOVA, and ANCOVA, the Random Effects Model, the Mixed Effects Model, Dunn and Clarck's *Z*_*j*_ statistic, serial correlations and Fisher's transformation of scores into *z* values. The benefit of these approaches lies in the facility to recognize and replicate them in most cases. The negative aspects reside in the difficulty to accomplish the statistical assumptions of every technique, especially the population normality of the distributions analyzed and the homoscedasticity assumption. In addition, when these techniques are employed in the generation of descriptive models (with limited possibility of inference) the objective remains far from what is required, which is nothing but a complex connectivity model. In addition, it is unusual to find these approaches in independence and self-correlation registers, which further hinders an optimal statistical approach.

We also found approaches to the general linear model with different analyses such as regressions and the discriminating analysis. Along these lines we found different choices from the simplest models [regression models with Ordinary Least Squares estimations (OLS)] to more complex ones based on Structural Equation Models or, occasionally, Path Analysis Models. Regarding the previous paragraph, data on the viability of linear and non-linear (frequently linearized) models is usually unavailable as regards model assumptions or the conditions of application. Most of these models are located within the domain of parametric statistical models, which leads to the same situation we described above. We noticed a scant description of assumptions and adopted modifications to ensure a correct parameter-estimation technique. Likewise, there are no acceptable approaches to the residuals generated by the models studied eventually. Neither are there assessments of the residuals' or the structural errors' independence from one another or between the signal values. To sum up, it seems inarguable that the authors should provide technical data on model fitting. This information would allow us to know in detail to what extent we find more descriptive models than the ones the authors intended to fit. One simple example will serve to illustrate this detail: it is unusual to report the value of the determination coefficient in uniequational and also multiequational models.

Of course, we can find the studies of dimensionality. As we mentioned before, these are widely applied in this field, and many researchers conducted the ICA. However, like in the situations described above, no special tradition exists to offer variability results in dimensionality reduction, either by ICA, as previously mentioned, or by Principal Component Analysis (PCA). In both cases, the selection of maximum explained variance vectors entails a process based on the normalization of the vectors representing the voxels' original values with regard to the ROI defined. Usually, there is no mention of the conditions of sphericity of the voxels selected or of the values that characterize the statistical viability of each ROI. The fact that ROI size is a determining factor to obtain a good solution (either in ICA or PCA) is unimportant. In smaller ROIs the conditions of unidimensionality are easier to obtain than in other techniques. Regarding these considerations, only seldom can we obtain values of explained variance in selected voxels. It could be irrelevant if variance values assumed by ROIs were high, as it usually happens but not always, and these cases go unnoticed because they are not reported.

Lastly, many authors chose for the analysis to conduct a model fit, which was mostly used to fit the data prior to the analysis. In this category, we found numerous examples, such as the Small World Analysis, the Gaussian Random field theory, ROC curves, behavioral vectors, the deconvolution analysis, the segmentation of brain regions, ALFF (Amplitude of Low Frequency Fluctuations) and fALFF (fractional Amplitude of Low Frequency Fluctuations) analyses, the spatial extent analysis, the estimation of deformation fields, the analysis of Regional Homogeneity (ReHo), the Granger Causality Analysis, the analysis of functional synchrony and DICCCOLs, the Dynamic Causal Model, and the Structural Equation Model. As we mentioned above, part of these approaches show the advantages and disadvantages of common linear models. Nevertheless, in our opinion, those are the most reasonable approaches to the study of connectivity, at least so far. The advantages are related to a fundamental issue, which is the fact that those techniques are devoted to the complexity of multiequational structures with a dynamic substratum. It is obvious that they are probably very far away from a feasible representational model, but they still imply a representational model that favors the networks and the comprehension of the structures. However, these techniques still need statistical complexity and, without some previous information, they are very difficult to replicate. For example, it is usual to omit information related to the estimation techniques and their defining values. Convergence criteria are not cited, neither are the values of initial solutions (if there are any) or the conditions of parametrization and reparametrization of estimations. Also, nothing is said about whether the authors opted for full or partial estimations, or whether they opted for colinearity robust estimations (like two-steps techniques) or for techniques based on parameter ponderation (like Weight Least Square). Finally, in this type of approach, the authors usually offer the final results in a simplified way completed with any kind of graph showing the activated areas in the brain, but usually no information is included about parameter intensity and what it means for the connectivity network.

It should be noted that it is common to use more than one analysis in a publication. Accordingly, many authors choose more than one test for each study. Specifically, in 32 papers, the researchers applied analytic techniques pertaining to different categories from the classification above.

Table 3 shows the frequencies of the main analyses and connectivity models proposed by the authors according to the classification used previously. The study of these papers about connectivity and MCI showed an obvious effect that confirms the scarcity of replicable works in an exact way, regarding the statistical models used. This situation, as mentioned above, is not exclusive of this domain but needs to be considered as an important problem to solve. In the following section we offer more details about this situation and some viable recommendations to solve it.

### Clinical results

In this section we intend to emphasize how the articles selected did not tackle, in general, the clinical aspects of the pathology. Only few works noticed the relationship between the estimated connectivity network and the intra- and between-groups effects; in the former case, in order to distinguish networks of specific population groups (for example, control vs. clinical group) and, in the latter case, for longitudinal course studies (for example, estimated networks in repeated measures paradigms). The results presented in these papers focus occasionally on secondary aspects such as those showing the effects of different degrees of severity (Miller et al., 2008) or those regarding the earliest stages of the pathology, when subjective and memory complaints are reported and, therefore, these are very subtle cognitive mistakes (Machulda et al., 2009).

It is true that, in some articles, we can find clinical implications of connectivity estimation. One example is the verification of the existence of compensatory mechanisms in different brain areas. This means that we can find some increased activity in particular regions of MCI patients as compared to normal adults to compensate the deficits in other areas (Krishnan et al., 2006; Bai et al., 2008). Another example is that increased activation in the hippocampus to solve memory tasks seems to predict early detection of Alzheimer's Disease (AD) (Dickerson et al., 2005; Mueller et al., 2012). However, in most of the publications we found the importance of the hippocampus regions on different aspects because it is one of the regions most involved in MCI pathology. Accordingly, changes in hippocampus activity are present in most MCI patients (Greicius et al., 2004; Johnson et al., 2004; Miller et al., 2008; Wang et al., 2011), converters and non-converters to AD, which make it difficult to conclude that changes in hippocampus activity could predict AD.

Therefore, we have seen that there is some clinical information in the articles included herein, but, in general, there is a scarcity of reported information on clinical consequences in the estimation of cognitive networks in MCI.

## Conclusions

In this article, we summarized the main features of the studies on functional brain connectivity through *f*MRI in MCI patients. Based on 79 publications, we described the most relevant elements, especially statistical models for the estimation of connectivity and some considerations on the clinical consequences of those studies.

Thus, most articles aimed to compare the functional brain connectivity network in MCI patients with that of Alzheimer's patients and/or adults with preserved cognition. Many authors opted for semantic or episodic memory tasks, although resting state designs are becoming more frequent. These allowed us to reduce the amount of interference in the data obtained. It seems reasonable to assume that fostering studies based on the DMN involves a simpler experimental system and fewer confounding variables than certain complex cognitive paradigms whose activation correlates are not clear. The brain region that is most frequently activated with significance on the connectivity network estimated in these articles is the hippocampus. However, in resting state designs there is a remarkable presence of other MTL and DMN regions, such as the inferior parietal lobe and the parahippocampal region. All of this is consistent with the definition of the DMN usually assumed. It is also the most studied region in the analysis of ROIs, both in the definitions before and after early connectivity analysis.

Undoubtedly we found the widest variety in the models of statistical estimation of functional brain connectivity chosen, as well as in the data analysis techniques used in relation to the general models. Most authors choose classic parametric strategies. Estimations based on Pearson's correlation were common, and so were estimations of partial and semi-partial correlations in order to isolate the effects as efficiently as possible. It seems evident that this procedure attempted to reduce the perverse effects of colinearity. However, within this category, we still found a large number of diverse analyses. Indeed, parametric statistical tests are frequent, and they are used to contrast specific data between samples and effects. This effect might be caused by the need to compare the different samples, because once they conducted an early connectivity analysis, many authors chose to present the results of the parametric tests in order to compare some specific data. The use of Student's *t* or ANOVA tests, both included in this category, is frequent in this type of paper, although they are not specific techniques for connectivity estimation. Accordingly, the difference in the values of specific ROIs among groups is often mistaken for differences in the structure and intensity of connectivity networks.

The results obtained in the different publications were diverse, which is consistent with the different goals predetermined by the authors. Still, we found a series of common features regarding functional brain connectivity in MCI patients. First, it was common to observe a reduced brain activity in many of the studied regions, such as the hippocampus or the lateral parietal cortex. Likewise, we also noticed increased brain activity in MTL regions during the task or in DMN regions during the resting state. The authors concluded that these were compensatory mechanisms; that is, in order to solve the task correctly, MCI patients required more activity and involvement from brain regions than the participants without cognitive deterioration. Therefore, the participants seemed to compensate for reduced connectivity in some regions by involving others, or even by increasing the activation within them. Other authors distinguished amnesic MCI patients from non-amnesic ones, and they concluded that the latter showed better performance and more preserved functional connectivity in general. Lastly, many articles proposed possible biomarkers of future cognitive deterioration or future progression to AD. The most frequent biomarkers found in those articles were changes in the DMN and hippocampus connectivity patterns, as well as those in the lateral temporal lobe and the posterior cingulate cortex. The images from *f*MRI were optimal for this type of study.

Taking into account the aforementioned information available in the articles included herein, we verified that the study of functional brain connectivity in MCI patients was a difficult subject to approach, as noted by Carp (2012) in studying general *f*MRI articles. The publications we consulted showed great variability concerning the connectivity model chosen. This fact may reflect the difficulty inherent in the choice of an ideal method to analyze brain connectivity. Given the remarkable differences between the techniques, it was difficult to make the right choice. In fact, in most cases, there seemed to be no theoretical justification. This situation also makes difficult to elaborate a meta-analysis of this topic, because of the disparity of methods and approaches, and the lack of statistical details. We can find some meta-analysis regarding clinical results, but it would be very difficult if we want to keep in mind statistical results and functional connectivity models found.

We did not find a previous article about this subject, so we consider that our summary provides a global idea about the state of statistical analysis in functional connectivity studies. We established several relevant aspects:

- The use of such dispersed statistical models prevents the comparison of results in an accumulative and integrated way. In fact, most papers do not justify their choice or establish clearly to what extent the assumptions of each model have been proven, assumed, or simply forgotten. The impossibility to compare results makes it very difficult to make suprastructural estimations of regular connectivity networks.
- Likewise, the complexity inherent to this type of statistical approach involves extreme difficulty in replicating analytical procedures. Not only are the data processing phases opaque, but some of the algorithms used are also underdeveloped. Except for the cases based on the estimations of correlations, it is truly difficult to strictly replicate some analytical procedures.
- In the case of the networks estimated in the papers under consideration, this situation is exactly the same and makes it unfeasible to conduct a simple accumulation of connectivity networks.
- In addition, this implies that each statistical model studies the conception of connectivity in a different way. Regardless of statistical matters, suffice it to say that the connectivity network based on simple parametric tests does not lead to the same result as Bayesian-based complex statistical tests. That being said, they coexist cooperatively in this study sector.
- An attempt to group the different statistical approaches only showed the disparity and lack of specificity we already mentioned. The classification we are proposing is just a general description that allows us to identify the primary areas of statistical interest.
- It is also evident that, in the case of MCI, the results were dependent on aspects outside the phenomenon at hand. The choice of technique has a strong influence on the result on which we base our choices of prediction and knowledge of the characteristics of these patients. Obviously, we should be alerted by the system's fragility.

We believe that this document could provide an idea of the complexity of article replication in functional connectivity studies of MCI patients. We considered that it is important to provide tools to the clinical professionals to better understand the MCI characteristics and elements to focus the diagnosis and treatment for every patient. It would be difficult if every article used a different approach in their analysis, so we aimed to highlight this point to the research community to improve the comparisons of results'.

Regarding the most clinical details about this pathology, our data shows that studies on connectivity networks have not provided, so far, relevant information for the applied field. Thus, we found little information on the CR protector effect on MCI appearance or on how networks show specific patterns for individuals with crystallized CR. Also, there is a lack of information about the use of networks for severity and risk prediction. Additionally, relevant information on the possible effect of frailty in the elderly for network estimation is missing, too. As we mentioned above, we can find some articles that provide possible biomarkers to detect MCI converters to AD, but these biomarkers seem to be far from an actual detection of AD converters. Evidently, this field is very complex and we are in a primary stadium of knowledge, which is insufficient to answer most of these unsolved questions and other characteristics of MCI. However, our paper does show that the problem comes before the study of clinical consequences as the use of technology and dispersed models causes difficulties in this matter. Consequently, we seem to be far from using these studies for clinical categorization in MCI patients.

As regards the differences in the information reported between articles, it could be useful to have guidelines to establish which information should be reported in an article on this topic in order to understand and replicate a study. Following the guidelines proposed by Poldrack et al. (2008) and, mentioned by Carp (2012) would facilitate the unification of information in the articles. Then, it would be possible to easily find the same information in every document and would clarify the statistical approach used. Also, it would allow for a global idea of this topic and would help in the elaboration on meta-analysis in this topic.

Furthermore, all things considered, it seems necessary to establish some recommendations for the MCI field and also for the general scope of connectivity estimation models from *f*MRI paradigms. They should, i) adopt techniques based on easily estimable statistics, such as correlation coefficients; ii) identify and describe all the phases in statistical analysis and to identify the tools used by its application; iii) provide instructions and analyses in an annex form, to be used in other data-bases or allow for replication; iv) clearly establish the correction mechanisms and their values in cases of classical corrections, such as the Bonferroni correction as well as more elaborated corrections, such as the False Discovery Rate (FDR); v) facilitate the fulfillment of model assumptions; vi) generate works with sufficient sample sizes to support a statistical model compatible with statistical predictor tools; and vii) clearly offer effectuated modifications or settings to the general estimation techniques.

In conclusion, we believe that the aforementioned aspects should be taken into account for future publications on functional brain connectivity in MCI patients. This way, better homogeneity would be achieved in brain connectivity study models and data analyses. This would make it possible to make comparisons between studies and the results would be more easily generalized.

### Conflict of interest statement

The authors declare that the research was conducted in the absence of any commercial or financial relationships that could be construed as a potential conflict of interest.
